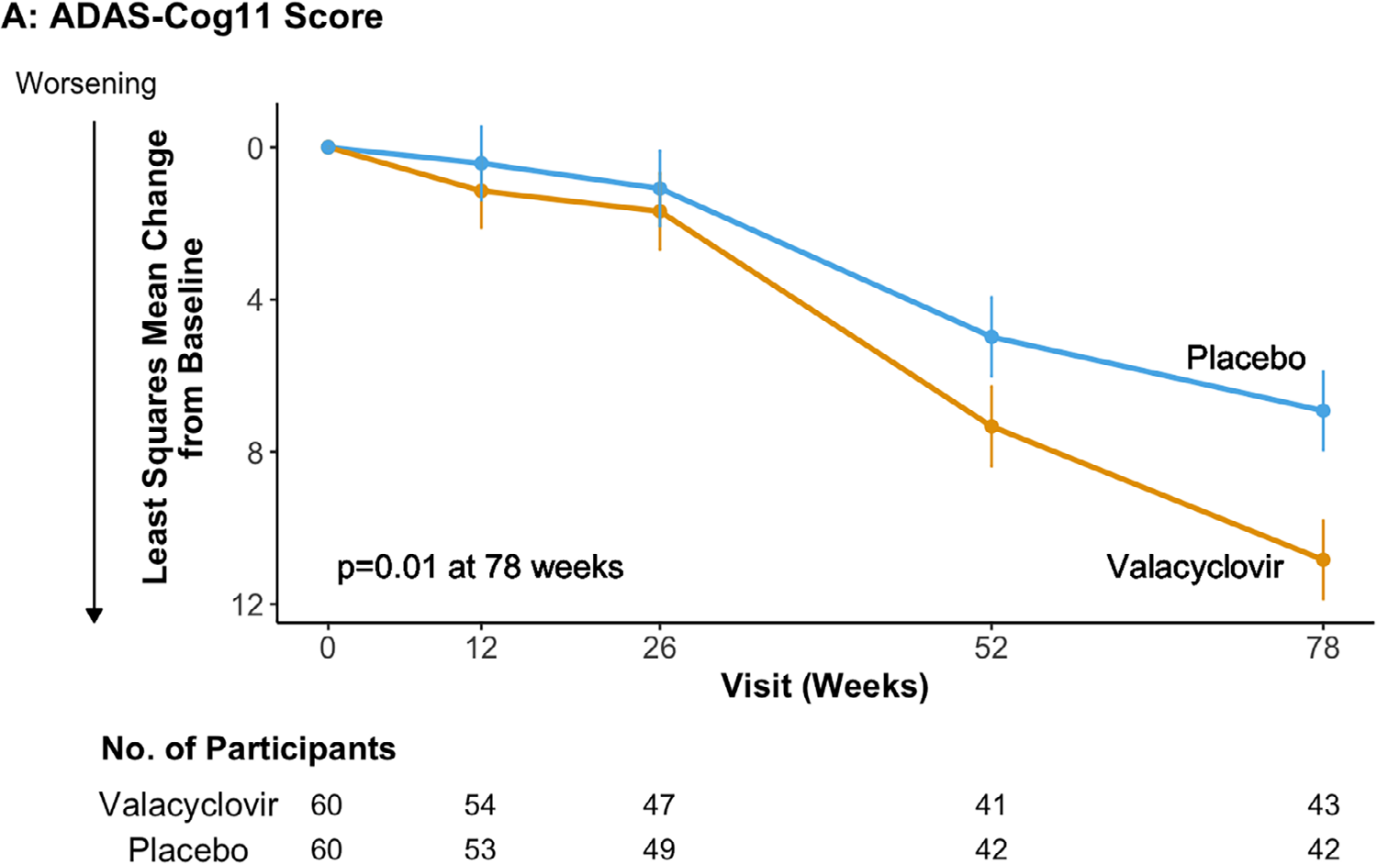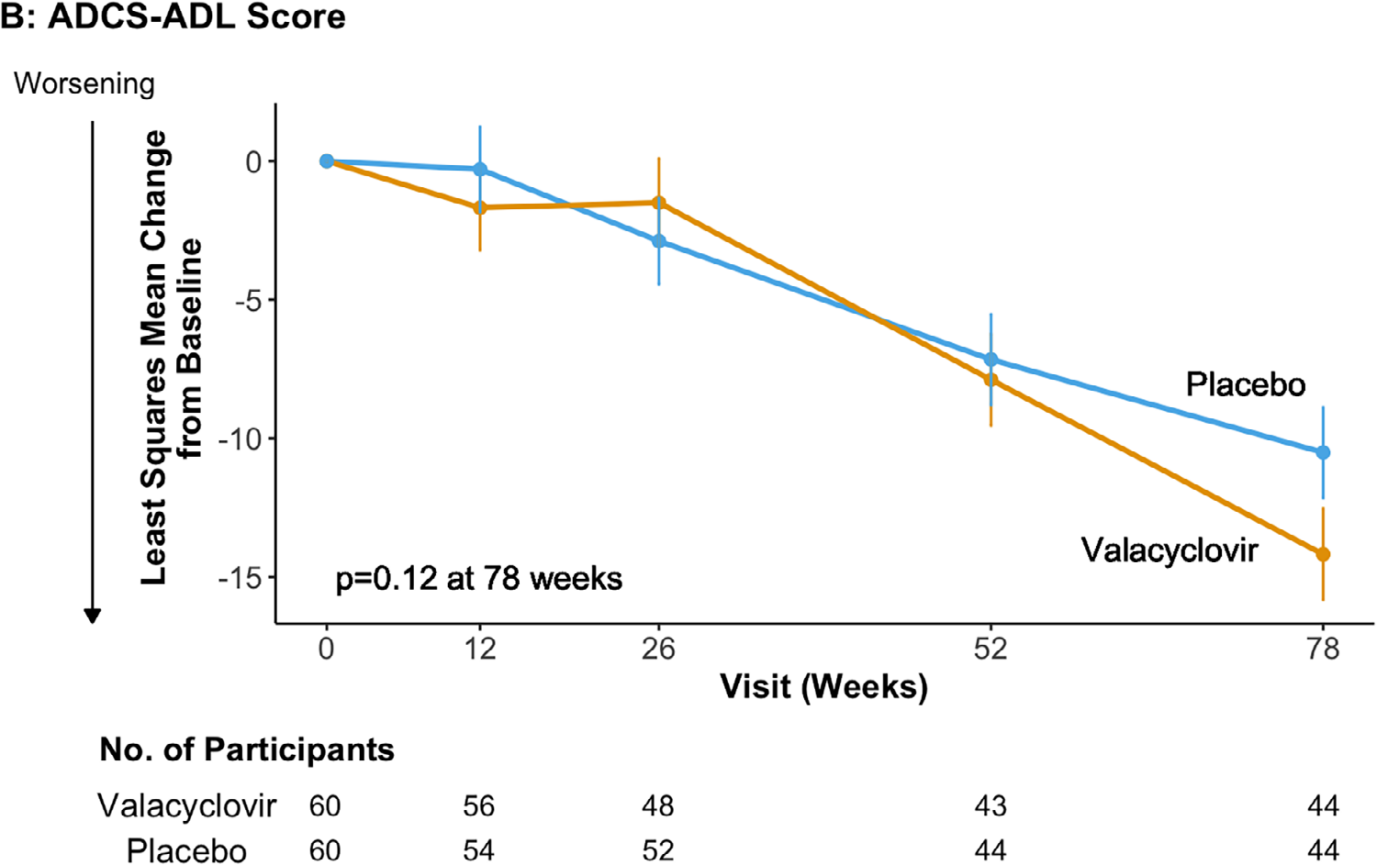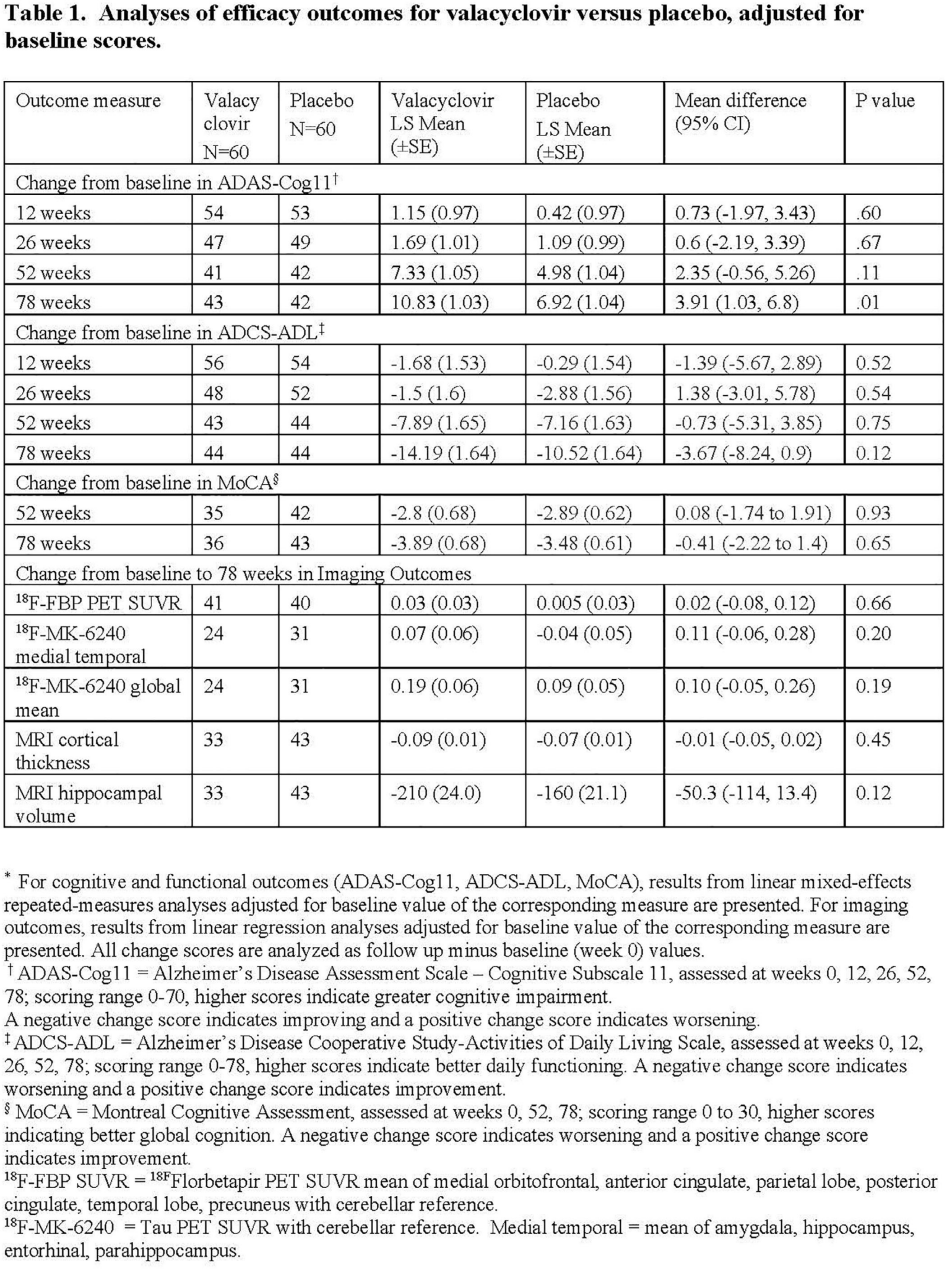# Antiviral therapy: Valacyclovir Treatment of Alzheimer's Disease (VALAD) Clinical Trial

**DOI:** 10.1002/alz70861_107951

**Published:** 2025-12-23

**Authors:** Davangere P. Devanand, Thomas Wisniewski, Qolamreza R Razlighi, Min Qian, Renjie Wei, Edward P Acosta, Karen L. Bell, Allison L Perrin, Edward D. Huey

**Affiliations:** ^1^ Columbia University Irving Medical Center, New York, NY USA; ^2^ Center for Cognitive Neurology, New York University Langone Health, New York, NY USA; ^3^ Alzheimer's Disease Research Center, New York University Langone Health, New York, NY USA; ^4^ Weill Cornell Medicine, New York City, NY USA; ^5^ Department of Biostatistics, Mailman School of Public Health, New York, NY USA; ^6^ Biostatistics, Mailman School of Public Health, New York, NY USA; ^7^ University of Alabama, Birmingham, AL USA; ^8^ Department of Neurology, Columbia University Vagelos College of Physicians and Surgeons, New York, NY USA; ^9^ Banner Health, Phoenix, AZ USA; ^10^ Department of Psychiatry and Human Behavior, Alpert Medical School of Brown University, Providence, RI USA

## Abstract

**Background:**

Infections may be etiologic or contribute to Alzheimer’s disease (AD) pathology. Evidence from neuroscience, epidemiology, and electronic health records data implicates herpes simplex viruses in AD, but controlled clinical trials of anti‐herpetic drugs have not been conducted.

**Method:**

In 120 participants at 3 U.S. sites with mild AD, or mild cognitive impairment (MCI) with positive AD biomarkers, the anti‐herpetic oral drug valacyclovir at 4 g/day, repurposed as an anti‐AD drug, was compared to placebo (60 valacyclovir, 60 placebo) in a Phase 2 randomized, double‐blind, parallel group trial. Seropositivity to herpes simplex virus (HSV) 1 or 2 was required. Acyclovir, the main metabolite of valacyclovir, was measured in plasma and in CSF in a subsample. The primary outcome was change in the 11‐item Alzheimer’s Disease Assessment Scale‐Cognitive Subscale (ADAS‐Cog11); secondary outcomes included the Alzheimer’s Disease Cooperative Study‐Activities of Daily Living (ADCS‐ADL) scale, Montreal Cognitive Assessment (MoCA), and neuroimaging conducted at baseline and 78 weeks utilizing ^18F^Florbetapir PET (mean of six brain regions), structural MRI indices, and ^18F^MK‐6240 PET tau imaging in a subsample.

**Result:**

Mixed model analyses adjusted for baseline score of the variable. The primary outcome, ADAS‐Cog11 score, showed greater worsening on valacyclovir than placebo at 78 weeks (LS Means difference 3.91, 95% CI 1.03 to 6.8, *p* =.01) with non‐significant differences at 12, 26 and 52 weeks in the same direction. ADCS‐ADL, the main functional measure, and other secondary clinical outcomes did not differ significantly between the treatment groups. Change in amyloid burden for ^18F^Florbetapir PET and tau for ^18F^MK‐6240 PET, and MRI cortical thickness and hippocampal volume, showed no treatment group differences. Mean plasma acyclovir concentration following oral doses of 4 g/day was 7140±5780 ng/ml (66 samples) and CSF acyclovir concentrations were 1260±460 ng/ml at week 12 (*n* =6) and 1270±1100 ng/ml at week 78 (*n* =3), which confirmed CNS penetration for valacyclovir. Adverse events did not differ significantly between valacyclovir and placebo.

**Conclusion:**

Valacyclovir was not efficacious as an antiviral treatment for participants with AD and herpes simplex virus seropositivity. The potential etiological role of herpes simplex viruses in AD needs re‐consideration.